# Unexpected localization of AQP3 and AQP4 induced by migration of primary cultured IMCD cells

**DOI:** 10.1038/s41598-021-91369-y

**Published:** 2021-06-07

**Authors:** Ralph Rose, Björn Kemper, Albrecht Schwab, Eberhard Schlatter, Bayram Edemir

**Affiliations:** 1 Department of Internal Medicine D, Experimental Nephrology, Münster, Germany; 2 Biomedizinisches Technologiezentrum der Medizinischen Fakultät Münster, Münster, Germany; 3 Institute of Physiology II, University of Münster, 48149 Münster, Germany; 4 Department of Medicine, Hematology and Oncology, Martin Luther University Halle-Wittenberg, Halle, Saale Germany

**Keywords:** Cell biology, Physiology, Nephrology

## Abstract

Aquaporin-2–4 (AQP) are expressed in the principal cells of the renal collecting duct (CD). Beside their role in water transport across membranes, several studies showed that AQPs can influence the migration of cells. It is unknown whether this also applies for renal CD cells. Another fact is that the expression of these AQPs is highly modulated by the external osmolality. Here we analyzed the localization of AQP2–4 in primary cultured renal inner medullary CD (IMCD) cells and how osmolality influences the migration behavior of these cells. The primary IMCD cells showed a collective migration behavior and there were no differences in the migration speed between cells cultivated either at 300 or 600 mosmol/kg. Acute increase from 300 to 600 mosmol/kg led to a marked reduction and vice versa an acute decrease from 600 to 300 mosmol/kg to a marked increase in migration speed. Interestingly, none of the analyzed AQPs were localized at the leading edge. While AQP3 disappeared within the first 2–3 rows of cells, AQP4 was enriched at the rear end. Further analysis indicated that migration induced lysosomal degradation of AQP3. This could be prevented by activation of the protein kinase A, inducing localization of AQP3 and AQP2 at the leading edge and increasing the migration speed.

## Introduction

Cell migration is a physiological part of body function that is relevant for every species from unicellular organisms to mammalians. It occurs in diverse physiological and pathophysiological processes including embryogenesis, immune response, wound healing and cancer metastasis^1,2^. There are several modes of cell migration such as amoeboid, mesenchymal and collective cell migration which share common basic underlying mechanisms^3^. At the beginning of the migration process, the cell must establish a polarity for defining the front and the rear of the cell. The lamellipodium at the front of the cell consists of a dense network of actin filaments. These actin filaments undergo a treadmilling mechanism of polymerization and depolymerization to push the cell body forward^2^. Moreover, the cell forms connections to the extracellular matrix (ECM). This interaction of the cytoskeleton with the ECM is mediated by specific actin-interacting proteins like integrin, paxillin, talin or vinculin that especially accounts for mesenchymal migration^4^. Cells encounter morphological changes during migration that are linked to changes in the cell volume. There is a continuous flow of ions and water across the cell membrane that is mediated by several ion channels and transporters^5^.

The transport of water requires the expression of members of the aquaporin (AQP) water channel family. So far 13 different members of the AQP family have been described; those function primarily as a water and solute channel and can be found ubiquitously in many cell types. They are involved in various functions i.e. brain swelling, neural functions and cell migration^6^. For example, the role of AQP1 in cell migration has been shown in AQP1 deficient endothelial cells. In these cells the migratory capability was significantly reduced^7^. These observations were also made in proximal tubule cells, where AQP1-deficiency caused impaired migration and decreased regeneration after acute ischemia^8^. In addition AQP2^9^, AQP3^10^, AQP4^11^, AQP5^12^ and AQP9^13^ were also found to play a significant role in cell migration. AQP2–4 are expressed in the collecting duct. They are specific markers of principal cells. AQP3 and AQP4 are constitutively expressed in the basolateral membrane of the principal cells^14,15^. AQP2 is localized in intracellular vesicles and its apical expression is induced by the action of the antidiuretic hormone vasopressin (AVP)^16,17^. Additionally it has been discovered that AQP3 is vasopressin responsive as well^18^. The binding of AVP to the AVP type-2 receptor (AVP-R) induces the activation of the adenylate cyclase, leading to increased cyclic adenosine monophosphate (cAMP), activation of the protein kinase A (PKA) and phosphorylation and translocation of AQP2 to the apical plasma membrane^16^. The cells in the inner medullary collecting duct (IMCD) are faced with a hyperosmotic environment which represents a stress component but is also of major importance for the urine concentration capacity. In this segment the fine tuning of the water reabsorption takes place in the IMCD and is facilitated by AQP2–4 with the hyperosmotic environment being the driving force^14^. The adaptation of the cells to the hyperosmotic environment is not only associated with the induction of so called osmoprotective genes, but also the expression of AQP2–4 is potently induced by hyper osmolality^19,20^. The tubular cells of the inner medullary collecting duct (IMCD) are at an increased risk of ischemic injury. This is due to a low pO2 milieu in the medulla, which renders these cells more prone to hypoxia^21^. Hypoxia and ischemia are well known causes of pre—and intrarenal acute kidney injury (AKI), which is accompanied with a high mortality, especially if it progresses to end stage renal disease (ESRD)^22^. The renal tubular cells possess the ability though to restore the damaged epithelium by collective cell migration^23^. This process is well studied in proximal tubule cells, but there are not many studies in the collecting duct.

Migrating cells develop a front to rear cell polarization axis. The front of the cells is oriented to direction of movement^24^. Water transport during cell migration was proposed as a major mechanism already 25 years ago with local cell swelling and shrinkage occurring at the cell front and rear end, respectively^25^. Recently, this mechanism was confirmed using microfluidic devices and called “Osmotic Engine” model^26^. Several studies have shown that AQPs are concentrated at the leading edge of the migrating cells^5^. A local osmotic gradient at the cell front leads to local water flow supporting the formation of protrusions and is enhanced upon expression of AQPs^7^. Water flow across the plasma membrane of the leading edge also provides an additional protrusive force^27^.

Hyper osmolality is a stress factor for cells and migration of cells in a hyperosmotic environment should represent a further challenge for the cells to develop the osmotic gradient that is necessary for migration and reduce the cell migration capability. On the other hand it induces the expression of AQP2–4, proteins that have been shown to promote cell migration.

Cell migration studies using primary IMCD cells, endogenously expressing AQP2–4, together with differences in osmolality of the cell culture conditions are missing.

We aimed to further elucidate the role of AQP 2–4 and the effect of hyper osmotic stress on cell migration in IMCD-cells for the first time. For this purpose, we have performed cell migration studies using primary IMCD cells. We further analyzed the effect of differences in osmolality on cell migration. We expected that osmotic stress would affect cell migration capability. Since the cells endogenously express AQP2–4 we expected enrichment of AQPs at the leading edge.

## Materials and methods

### Primary inner medullary collecting duct cells

The primary cultures of the IMCD- cells have been prepared as previously described^19,28^. For the cell culture medium, Dulbecco’s modified Eagle’s medium (DMEM) containing 100 IU/mL penicillin and 100 μg/mL streptomycin, 0.2% glutamine, 1% non-essential amino acids, and 1% ultroser (BioSepra Inc., Marlborough, MA, USA) was used. The medium osmolality was increased to 600 mOsmol/kg by the addition of 100 mM NaCl and 100 mM urea. Osmolality was checked using an osmometer (Knauer, Berlin, Germany)^29^. For each experiment a new primary culture was prepared. One day before the extraction of the kidneys the cell culture dishes were coated with 2 mL collagen type IV and stored overnight at 4 °C. The next day the collagen was removed, and the slides were washed two times with sterile PBS. The surface of the dishes should be dry to ensure sufficient attachment of the cells. The kidneys of female Wistar rats were used for the extraction of the IMCD-cells. Two kidneys were used to cover an area of 18 cm^2^ with cells. The rats were sacrificed, and the kidneys removed, followed by isolation of the papilla, including the inner medulla, which can be distinguished from the rest of the kidney through its white color. The medullas were chopped into pieces with a scissor and transferred into the enzyme solution for digestion. The cell suspension was placed into the water bath and incubated for approximately two hours at 37 °C and continuous shaking at 240 rpm. After incubation the cell suspension was homogenized by pipetting 50 times and centrifuged at 300 × *g* for 5 min. The supernatant was removed, and the cell pellet was washed with 30 mL sterile PBS at room temperature and centrifuged a second time. Afterwards the cells were suspended in 600 mosmol/kg medium and allocated to the coated dishes. The cells were cultivated in 600 mosmol/kg medium for the initial 24 h. After 24 h the medium was replaced by the same medium or switched to 300 mosmol/kg medium. In further experiments only cells were used that were at least cultivated for 5 days at 300 or 600 mosmol/kg. Experiments were approved by the governmental-committee on animal welfare (Landesamt für Natur, Umwelt und Verbraucherschutz NRW (LANUV), Münster; 84-02.05.20.11.256) and were performed in accordance with national animal protection and in compliance with the ARRIVE guidelines^30^.

### Wound healing assays

Prior to the migration experiments the MEM medium was prepared as indicated above, but without Ultroser. The serum deprivation was necessary for stimulation experiments to avoid prior activation of cell receptors. HEPES-buffer was used to maintain a physiological pH at 7.4. The experiments were performed in collagen type IV coated 35 mm dishes. A scratch wound was made using a 20 µL pipette tip. After the scratch was induced, the cells were washed with PBS and the medium was replaced by HEPES buffered MEM. Four different medium conditions were used. The medium of cells initially cultivated at 300 mosmol/kg was replaced by 300 mosmol/kg and of cells initial cultivated at 600 mosmol/kg by 600 mosmol/kg medium, respectively. During condition 3 and 4 we acutely challenged the cells initially cultivated at 300 mosmol/kg with 600 mosmol/kg medium and cells initially cultivated at 600 mosmol/kg with 300 mosmol/kg medium during the migration period.

Cell migration was monitored as previously described^31^. The dishes were placed immediately into the preheated chambers of the microscopes and the scratch was oriented parallel to the upper and lower image border.

The migration was recorded in 5 min intervals for 15 h. Images were taken with a Zeiss Axiovert 40c microscope. Cell migration was quantified as the movement of both wound borders into the wounded area within 15 h. The wound border was defined as the leading first cell row at the beginning and the end of the migration. Measuring the distance between the wound edges at t = 0 h and t = 15 h allowed to calculate the net distance covered by both wound edges and derived thereof the speed (µm/h) of the wound closure. We used Fiji image analysis software to display the wound healing assay and for their quantitative analysis^32^. Movies were also prepared using the ImageJ software.

### Immunofluorescence

Proteins that are involved in migration were analyzed after performing a scratch assay as described above. The IMCD cells were seeded onto collagen type IV coated glass cover slips in a 24 well plate. The cells were fixed in PBS containing 4% paraformaldehyde (PFA) for 20 min after a scratch wound has been induced. The cells were washed with PBS (3 × 15 min) and then permeabilized in 0.1% Triton X 100 for 5 min and washed a second time with PBS (3 × 15 min). The cells were incubated with a blocking solution, containing 0.3% fish skin gelatin in PBS for 20 min at 37 °C. Then the cells were incubated overnight at 4 °C with the primary antibodies. The next day the cells were washed three times with PBS and incubated with an Alexa-488 or Alexa-594 labeled anti-rabbit or anti-mouse IgG antibody (Thermo Fischer Scientific, Waltham, MA, USA), depending on the species of the first antibody. In order to label the actin filaments, the cells were incubated with Alexa 488 or 594 labeled phalloidin (Thermo Fischer Scientific). The nucleus was stained by DAPI. Cells were washed one last time with PBS and mounted with Immu-Mount (Thermo Fischer Scientific). Images were taken with a Zeiss Axio Observer Z1 microscope (Zeiss, Oberkochern, Germany). The Antibodies for AQP2–4 were obtained from Alamone Labs (Jerusalem, Israel). The NHE1 antibody from BD Biosciences Pharmingen (San Jose, CA, USA). All other antibodies were obtained from Cell Signaling Technology (Boston, MA, USA). Signal intensities of cell profiles were generated using Fiji image analysis software^32^. Also the cell areas were analyzed with the Fiji image analysis software.

### Western Blot analysis

Western blots were performed as described before^33^. Briefly, the cells were lysed using a modified Laemmli-buffer for 24 h under constant shaking. A 4–20% polyacrylamide-gel was loaded with the samples and a size marker Pageruler Prestained (Thermo Fischer Scientific) and the proteins were separated by gel electrophoresis. The proteins were transferred from the gel to a polydivinyldifluoride-membrane (PVDF-Membrane, Merck Darmstadt, Germany). The membrane was cut prior to hybridization with the antibodies to simultaneously incubate different antibodies of similar molecular weight. Unspecific binding of the primary antibody was blocked before by incubation with 5% BSA in TBST for 30 min. Membranes were subsequently incubated with primary antibodies in a 1:1.000 dilution. Antibodies raised against AQP2–4 were obtained from Alomone Labs (Jerusalem, Israel)^34^. Antibodies against NHE-1 were obtained from BD Biosciences Pharmingen (San Jose, USA)^35^ The reagent Lumilight was added to the membrane for 10 min and the signal was detected with the Lumi Imager F1 (Roche Diagnostics, Basel, Switzerland). In order to ensure equal loading, the membrane was stripped (30 min/60 °C) using stripping buffer (15 g/L glycine, 1 g/L sodium dodecyl sulfate, 10 mL/L Tween 20, pH to 2.2) and incubated with an antibody directed against glyceraldehyde 3-phosphate dehydrogenase (GAPDH). Lumianalyst software (Roche, Basel, Switzerland) was used for quantification of specific bands.

### Quantitative phase imaging-based cell analysis with digital holographic microscopy

Time resolved quantitative phase imaging (QPI) analysis of cellular volume and the covered area of IMCD cells was performed by label-free digital holographic microscopy (DHM). Experimental investigations were conducted either with an inverted iMIC microscope (Till Photonics, Gräfelfing, Germany) with an attached DHM module, and an incubator (Solent Scientific Ltd., Segensworth^36^, or with a Zeiss Axio Observer microscope modified for DHM, where temperature stabilization was achieved by an ibidi HT200 heating chamber (ibidi GmbH, Munich, Germany)^37^. In both DHM setups the coherent light source for hologram recording was a frequency doubled solid state laser (emission wavelength of λ = 532 nm). The reconstruction of quantitative DHM phase contrast images from the digitally captured holograms was performed by spatial phase shifting-based algorithms as reported earlier^38,39^ with custom build software. For QPI-based analysis, IMCD cells were cultivated confluently in collagen coated μ-dishes (ibidi GmbH, Munich, Germany). Cell free wound areas were created by scratches with a pipette. For DHM observation standard polymer Petri dish caps were replaced by glass lids (ibidi GmbH, Munich, Germany). Digital holograms of the wound areas at 300 mOsmol and 600 mOsmol were recorded every, 2 min (iMIC microscope, Zeiss EC Plan-Neofluar 10×/0.3) or 3 min (Zeiss LD Acroplan 20×/0.4 Korr) for a period of 3 h. Two independent DHM analyses were performed for each experiment.

### Statistics

The statistical analysis of the data was carried out using the program Graph Pad Prism 5.3 (Graph Pad Software Inc., San Diego, CA, USA). Statistical significance was analyzed by one-way ANOVA with Tukey’s post-test and two-tailed t-test for data following normal distribution. A significant result was set at *p < 0.05 and **p < 0.001. All values are presented as mean ± SEM.

## Results

### The expression of AQP3 and AQP4 is induced by the extracellular hyper osmolality

The primary cultured IMCD cells have been extensively used to study the vasopressin mediated translocation of AQP2 to the plasma membrane^33,40,41^. The major advantage of this model is the endogenous expression of AQP2. The expression of AQP2 in this model was increased by the hyperosmolar medium^20^. We have recently shown that hyper osmolality not only induces the expression of AQP2 but also the expression of other major principal cell markers like AQP3-4, the urea transporter Slc14a1 or vasopressin type 2 receptor mRNAs^19^.

**Figure 1:**
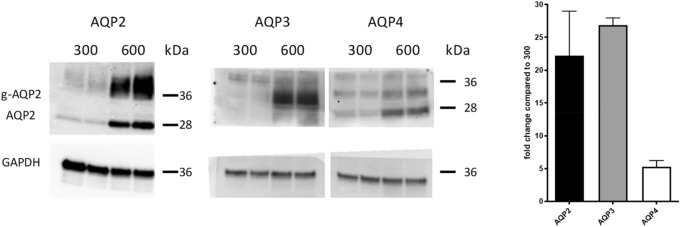
Protein expression of AQP2, AQP3 and AQP4 increases at higher osmolality. IMCD-cells have been cultivated in medium of 300 and 600 mosmol/kg. Proteins have been isolated and Western Blot was performed with antibodies directed against AQP2, AQP3, AQP4 and Glyceraldehyde 3-phosphate dehydrogenase (Gapdh). B: Densitometric analysis of AQP2, AQP3 and AQP4 protein level expression in cells cultivated at 600 mosmol/kg (relative to 300 mosmol/kg). The membranes were cut before hybridization with the first antibody. After stripping of the first antibody the membranes were probed with an anti GAPGH antibody. All values are means ± SEM (n ≥ 2). Uncropped images are provided as Supplementary Fig. 1.

We performed Western blot analysis to confirm the effects also on protein expression of AQP3 and AQP4 in this cell model. We used cells that have been cultivated either at 300 or 600 mosmol/kg and analyzed the expression by Western blotting (Fig. 1).

**Figure 2:**
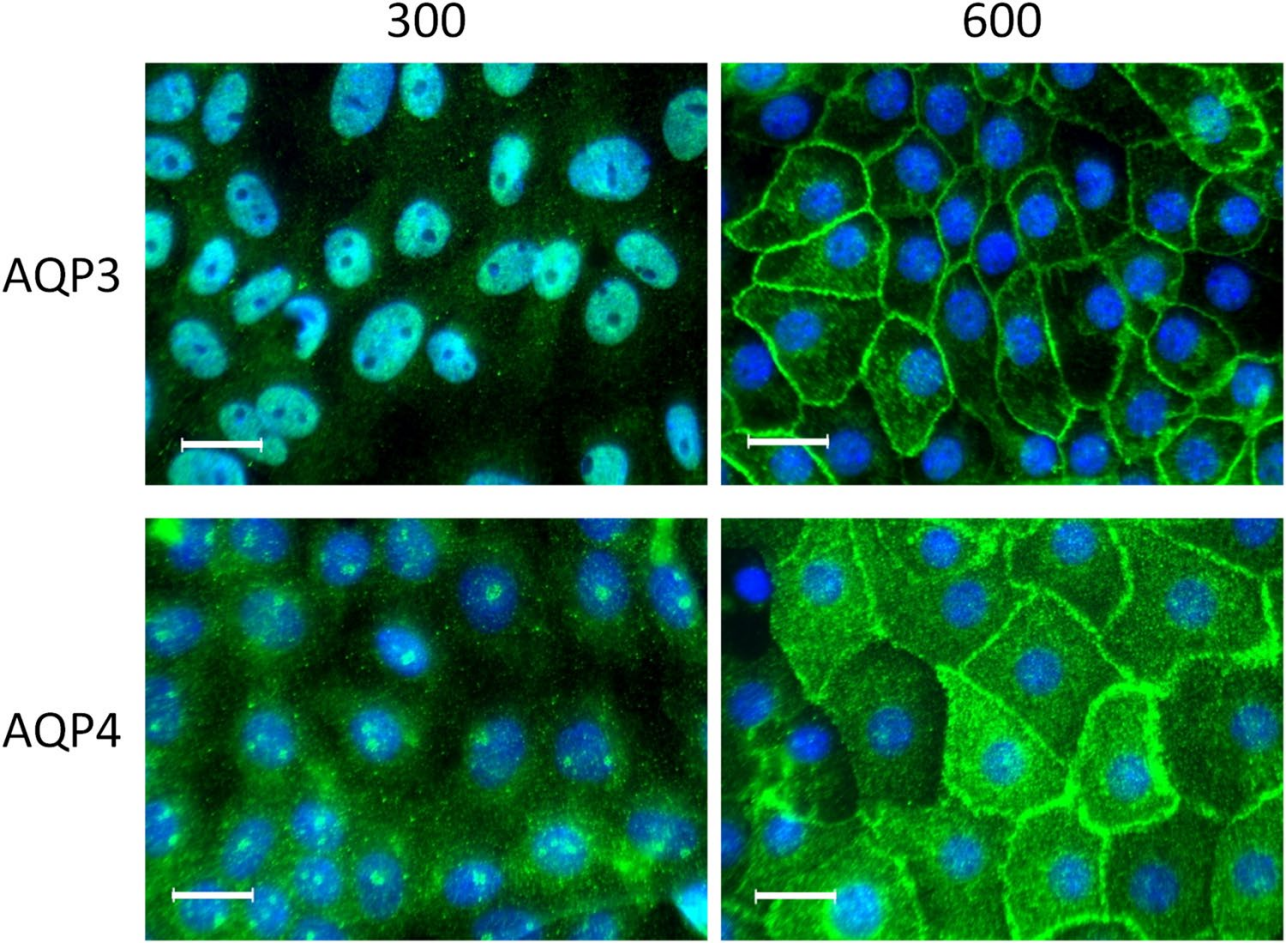
AQP3 and AQP4 are localized at plasma membrane. IMCD-cells were cultivated at 300 (right panel) and 600 mosmol/kg (left panel). AQP3 and AQP4 were detected using specific primary antibodies. Signals were detected using secondary Alexa-Fluor-488 labeled antibodies. The cell nucleus was stained with DAPI (blue). Images are representative for up to ten independent experiments. Scale bar, 20 µm.

The results showed that the protein expression of AQP3 and AQP4 was increased 26- and 5-fold, respectively, in IMCD cells cultivated at 600 mosmol/kg compared with those cultivated at 300 mosmol/kg. Thus, the increased mRNA expression of AQP3 and AQP4 is associated with an increased protein expression^19^. Immunofluorescence experiments further confirmed the differences in expression. As mentioned before, AQP3 and AQP4 are constitutively expressed in the plasma membrane of the principal cells of the collecting duct. While AQP3 and AQP4 were not detectable at 300 mosmol/kg, they are, as expected, enriched at the plasma membrane at 600 mosmol/kg (Fig. 2). Profile plots showing the signal distribution are provided as Supplementary Fig. 2.

These results showed that the primary cultured IMCD cells are enriched for principal cells. Therefore, they were a suitable model to analyze the role of AQP2–4 in cell migration but differences in osmolality have to be considered within the experimental setup.

### Osmolality has a major impact on cell morphology

**Figure 3:**
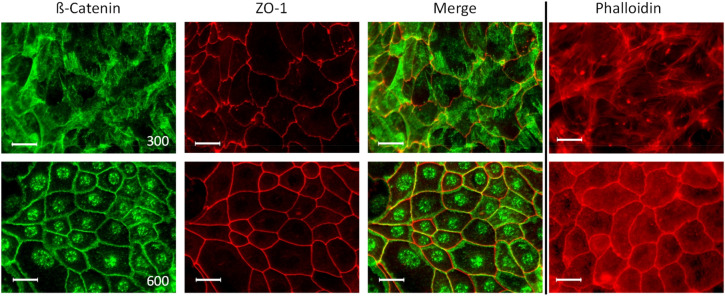
Osmolality has an impact on cell morphology. IMCD-cells were cultivated at 300 (upper panel) and 600 mosmol/kg (lower panel) and staining for β-catenin, Zo-1 and actin was performed using specific antibodies or phalloidin (for actin) respectively. Secondary Alexa-488 or Alexa-568 labeled antibodies or Alexa-568 labeled phalloidin were used for visualization. Scale bar = 20 µm.

We have recently shown that osmolality induces and suppresses the expression of thousands of genes^19^. Consequently, we tested how osmolality affects the morphology of the IMCD cells and stained IMCD cells that were cultivated at 300 or 600 mosmol/kg with different cellular markers. We used antibodies against zona occludens 1 (ZO1; for tight junctions), beta catenin (adherens junctions) and phalloidin (actin cytoskeleton; Fig. 3).

The staining for beta catenin in cells cultivated at 300 mosmol/kg demonstrated a diffuse staining pattern and the cell borders were hard to be distinguished. This is also evident for the phalloidin staining. At 300 mosmol/kg the actin cytoskeleton exhibits a dense network of stress fibers. The staining pattern completely changed in cells cultivated at 600 mosmol/kg. Beta catenin is localized in the nucleus and is also enriched at the cell–cell contacts. The ZO1 staining is uneven in cells cultivated at 300 mosmol/kg and in cells cultivated at 600 mosmol/kg the ZO1 staining shows the cobblestone like distribution that is typical for epithelial cells. Also the actin staining shows massive changes since and we observed an accumulation of actin at the cell–cell contacts. This is also evident in migrating cells (Supplementary Fig. 3). The ZO1staining was used to calculate the area that is covered by a cell. The analysis showed no significant differences between cells cultivated at 300 and 600 mosmol/kg (Supplementary Fig. 4).

### Effect of osmolality on migration of IMCD cells

**Figure 4:**
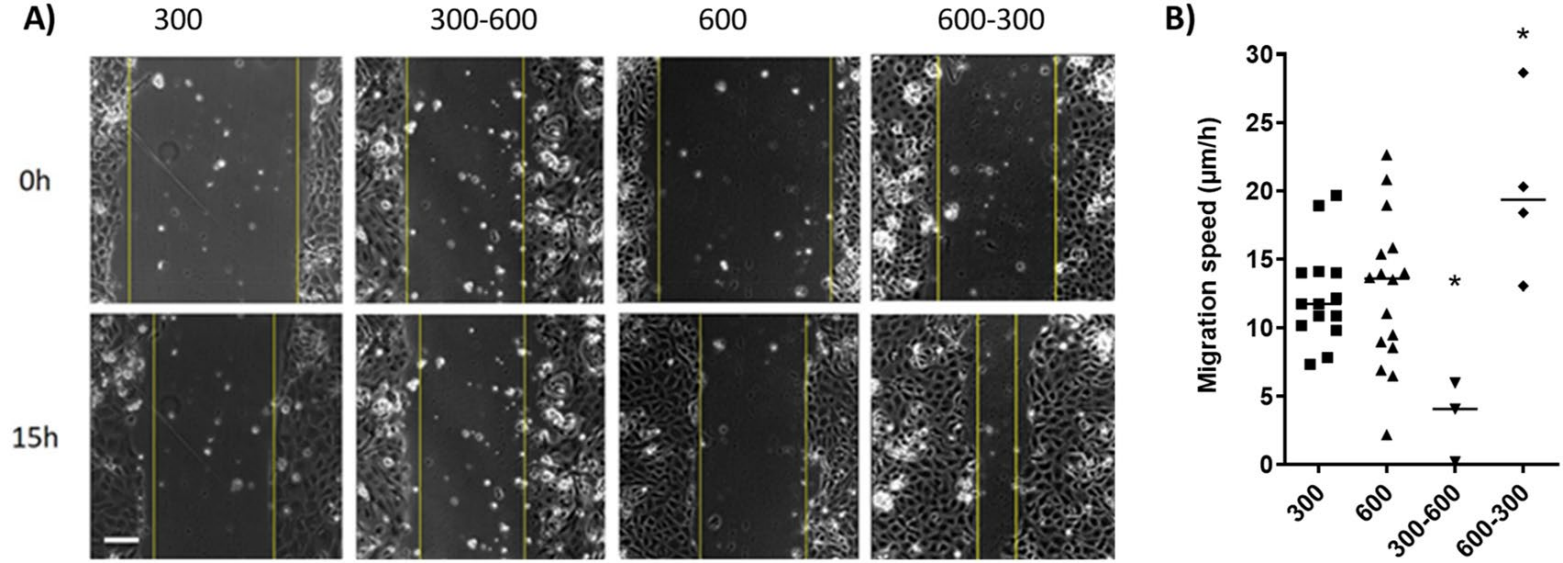
Primary cultured IMCD cells show a collective cell migration behavior. (**A**) Wound healing assays were performed with IMCD-cells that were cultivated at 300 or 600 mosmol/kg and in cells where the osmolality was acutely increased from 300 to 600 mosmol/kg (300–600) or decreased from 600 to 300 mosmol/kg (600–300). A scratch was induced using a pipette tip. The migration was observed for 15 h. During migration the cells were cultivated in DMEM without serum. (**B**) The sum of the migration speed from both migration fronts was calculated and is presented as mean µm/h ± SEM. Statistically significant differences are marked by *. ANOVA, p < 0.05, n ≥ 14 (for 300 and 600) and n = 3 for 300–600 and n = 4 for 600–300. Scale bar 150 µm.

In the next step we tested whether the IMCD cells were able to migrate. To this end we performed scratch/wound healing assays. To our knowledge this is the first time that this has been done in primary cultured IMCD cells. Figure 4 shows that the IMCD cells are able to migrate and that they show a collective cell migration behavior. We have shown that AQP2–4 expression is induced by hyper osmolality and also that the cell morphology including actin cytoskeleton is affected by the cell culture conditions. The actin cytoskeleton plays a major role in the migration process and the contribution of AQPs to cell migration has also been shown. Thus, we expected differences in the migration speed. In the next step we analyzed how the migration behavior of the primary IMCD cells is affected by the environmental osmolality. For this reason we used IMCD cells cultivated at 300 or 600 mosmol/kg and performed wound healing assays. Using a second approach cells initially cultivated at 300 mosmol/kg were acutely challenged during the migration period with 600 mosmol/kg medium and cells initially cultivated at 600 with 300 mosmol/kg medium. Movies of the migrating cells are provided as supplement.

**Figure 5:**
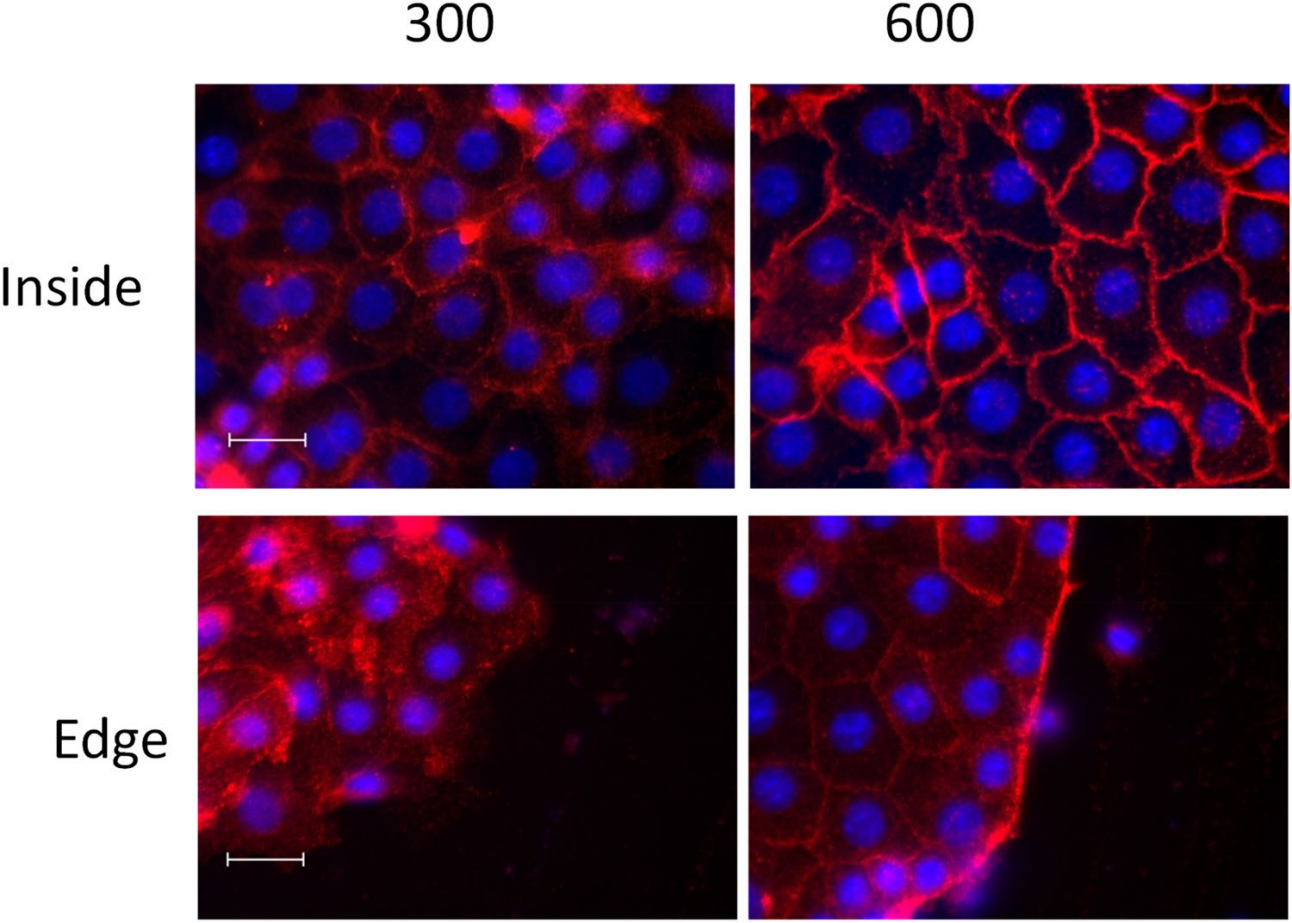
Localization of NHE1 in migrating IMCD-cells at different osmolality. IMCD-cells were cultured at 300- and 600 mosmol/kg. A scratch was induced and the cells were further cultivated for four hours. After that the cells were incubated with NHE1- antibody and Alexa-594 labeled secondary antibody. Images were taken from the leading edge of migrating cells and from the inside of the cell monolayer. Profile plots are provided as Supplementary Fig. 5. Scale bar, 20 μm.

The results showed that the hyper osmolality per se did not affect the migration speed of cells cultivated at 300 or 600 mosmol/kg (Fig. 4). An acute change of osmolality however, changed the cell speed remarkably. When we switched the osmolality from 300 to 600 mosmol/kg, thus inducing an acute hyperosmotic shock, the cell speed decreased significantly by about 70%. On the other side inducing an acute hypoosmotic shock, by changing the osmolality from 600 to 300 mosmol/kg increased the cell speed by approximately 40%. The results indicate that beside the hyper osmotic stress those cells are capable to generate an osmotic gradient at the leading edge that is necessary for cell migration. The Na+/H+ exchanger 1 (NHE1) mediates Na+ uptake in the leading edge supporting water flux thereby facilitating leading edge protrusion^26^. In the next step we have analyzed NHE1 expression in the cells (Fig. 5).

In contrast to cells cultivated at 300 mosmol/kg, NHE1 is enriched at the leading edge of the migrating cells cultivated at 600 mosmol/kg. Profile plots showing signal intensities are provide as Supplementary Fig. 6. Increased NHE1 activity could support cell migration even under hyperosmotic stress. The expression of NHE1 on the protein level is also induced by hyper osmolality (Supplementary Fig. 6).

The primary cultured IMCD cells were fully differentiated and did not proliferate. We have used 5′-bromo-2′-deoxyuridine (BrdU) incorporation assay to analyze whether the migration was mediated by increased proliferation. The results showed no BrdU staining in cells cultivated at 300 mosmol/kg and BrdU positive cells at 600 mosmol/kg (Supplementary Fig. 7).

Since the hyperosmotic environment induces DNA damages, the positive BrdU staining might be due to DNA repair mechanisms. No enrichment of BrdU staining is evident at the leading edge. Also the movies showed that coverage of the wound is not mediated by proliferating cells.

Cells were observed with quantitative DHM phase contrast as described in “Quantitative phase imaging-based cell analysis with digital holographic microscopy” and we have measured the covered area of the first row of cells (the results are shown in Supplementary Fig. 8). The covered area per cell increases. Interestingly this effect was much bigger in cells cultivated at 600 mosmol/kg. Furthermore, we the cellular volume increased in cells cultivated at 600 mosmol/kg (Supplementary Fig. 8).

### Migration alters the localization of AQP3 and AQP4

**Figure 6:**
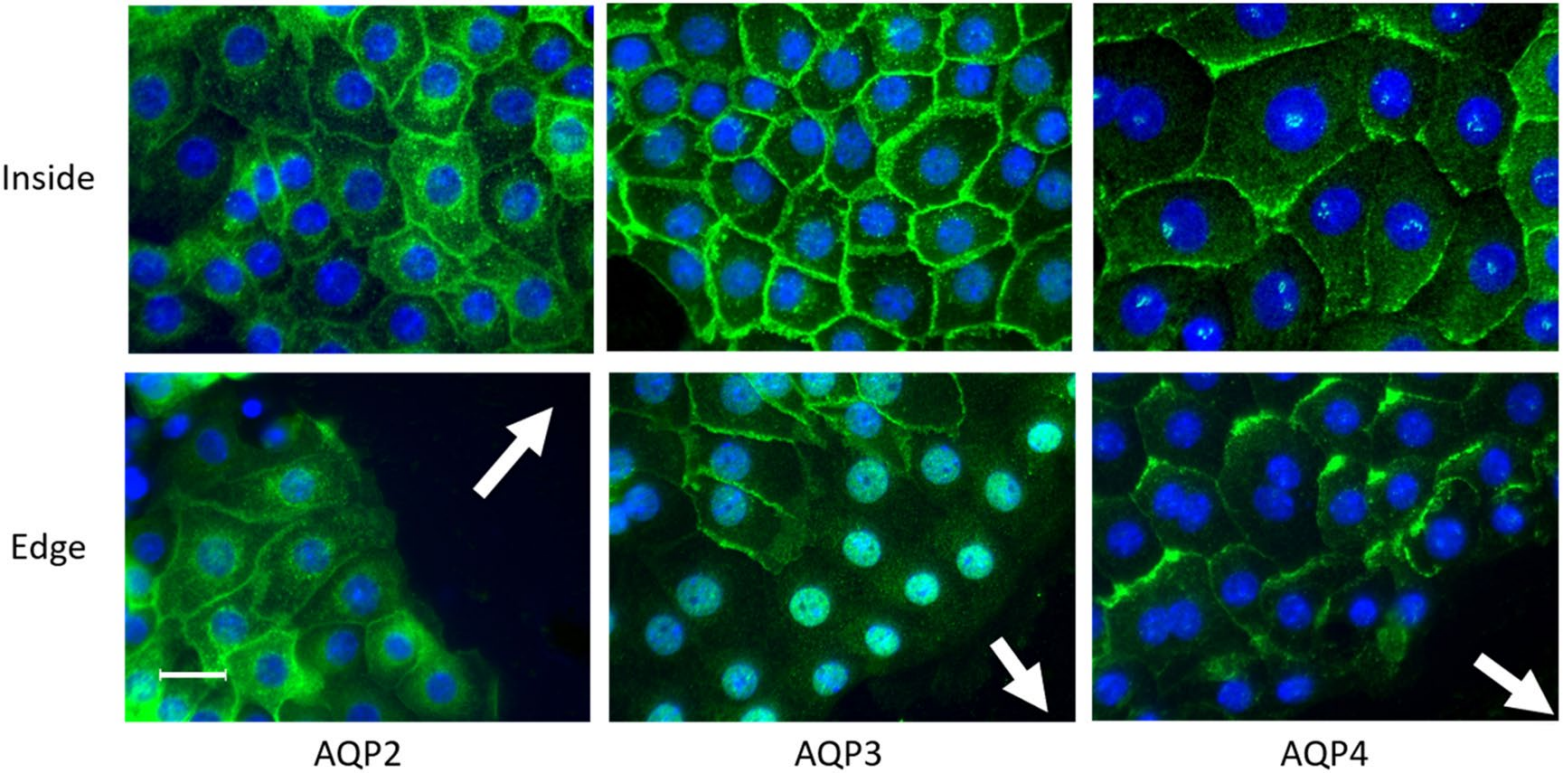
Migration influences the localization of AQPs. Wound healing assays were performed with IMCD-cells cultivated at 600 mosmol/kg. A scratch was induced and after 4 h the cells were stained for AQP2–4 using specific antibodies. For the AQP2 staining the cells were incubated with dbcAMP to induce membrane localization. Alexa-488 labeled secondary antibodies were used for visualization. The nuclei were stained with DAPI. The upper row show cells away from the wound. The lower rows show cells at the leading edge. Scale bar = 20 µm, representative image chosen from 6 to 9 independent experiments.

In the next step we analyzed whether the migration of the cells leads to the accumulation of the AQPs at their leading edges. Since AQP2–4 were only expressed under hyperosmotic conditions we have only used cells that were cultivated at 600 mosmol/kg. A scratch was induced and the cells were cultivated for additional 4 h and then used for immunofluorescence staining. The localization of AQP2 in the plasma membrane was induced by the addition of dibutyryl cyclic adenosine monophosphate (dbcAMP) during the 4 h period. The immunofluorescence analysis showed that AQP2–4 were, as expected, localized at the plasma membrane in areas away from the scratch (Fig. 6).

There was a slight staining for AQP2 at the leading edges of the cells in dbcAMP stimulated cells. This was not the case in unstimulated cells. Interestingly AQP3 staining disappeared in the first 2–3 rows of the migrating cells. The staining for AQP4 also showed no accumulation at the leading edges of the cells but AQP4 is accumulated at the rear edges of the migrating cells. The profile plots showing signal distribution are provided as Supplementary Fig. 9.

### Migration leads to lysosomal degradation of AQP3

**Figure 7:**
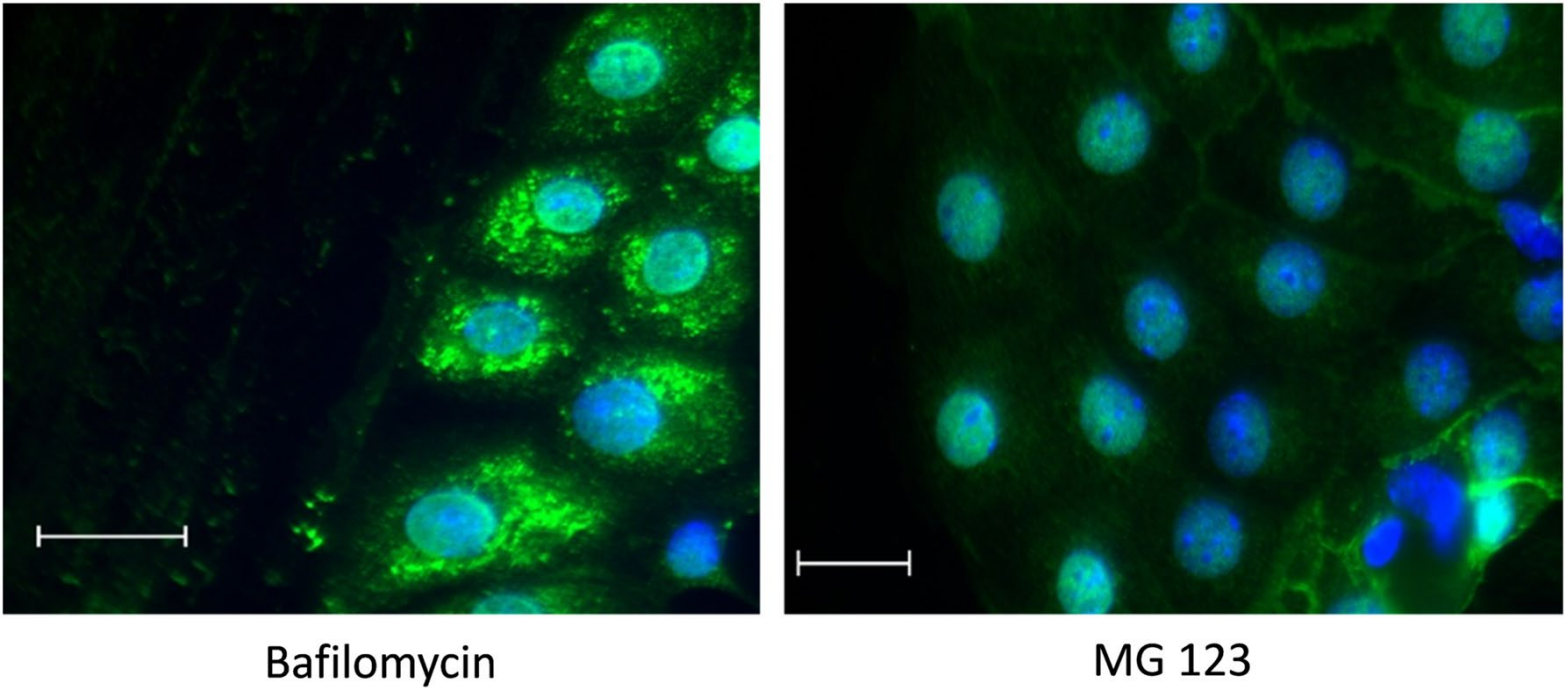
Intracellular accumulation of AQP3 after bafilomycin treatment. Wound healing assays were performed with IMCD-cells cultivated at 600 mosmol/kg. A wound was induced and the cells were treated with V-ATPase activity inhibitor bafilomycin (100 nM) or the proteasomal activity inhibitor MG132 (2 µM). After 4 h the cells were stained for AQP3 using specific antibodies. Alexa-488 labeled secondary antibodies were used for visualization. The nuclei were stained with DAPI. Scale bar = 20 µm, representative image chosen from 2 independent experiments.

To analyze why AQP3 disappears at the leading edge we treated the cells with inhibitors that block the degradation of proteins. We used bafilomycin to block the activity of the vacuolar H^+^-ATPase and thereby lysosomal degradation and MG132 to block proteasomal degradation (Fig. 7).

The treatment with bafilomycin induced the accumulation of AQP3 in intracellular compartments. The treatment with MG132 had no influence on the localization and AQP3 still disappeared within the first 2–3 rows of cells (Fig. 7). Profile plots showing signal intensities are provided as Supplementary Fig. 10. Treatment with bafilomycin also induced accumulation of AQP3 in intracellular structures in cell layer without introduction of a scratch (Supplementary Fig. 11).

### dbcAMP prevents migration associated disappearance of AQP3

**Figure 8:**
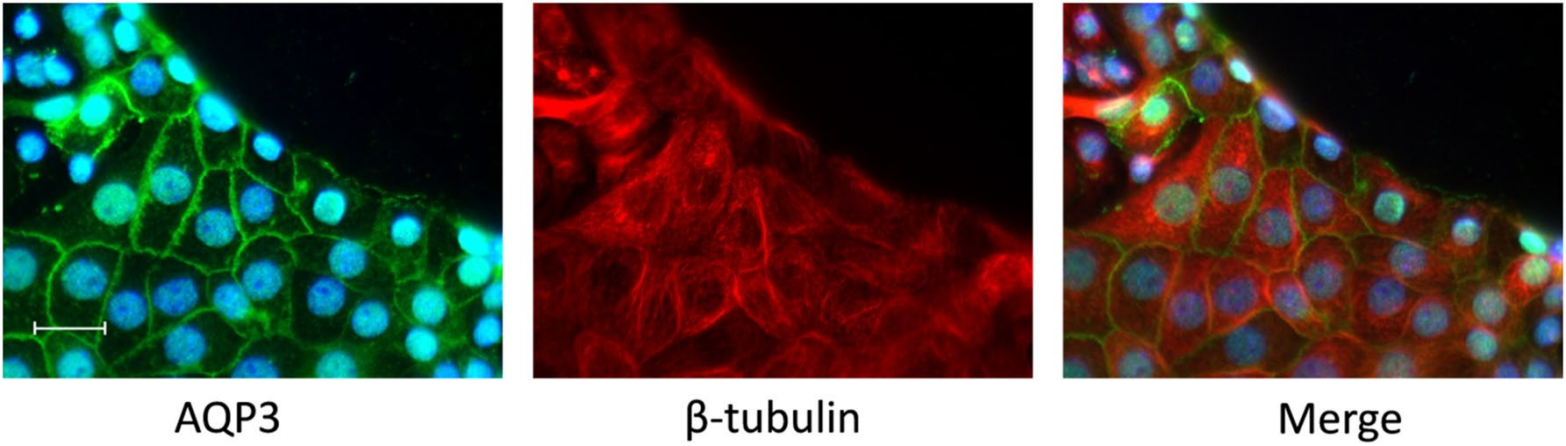
The treatment with dbcAMP prevented the degradation of AQP3. Wound healing assays were performed with IMCD-cells cultivated at 600 mosmol/kg. A wound was induced and after 4 h the cells were stained for AQP3 and beta tubulin using specific antibodies. During this period of time the cells were treated with dbcAMP (250 µM). Alexa-488 (green) and Alexa-568 (red) labeled secondary antibodies were used for visualization. The nuclei were stained with DAPI. Scale bar = 20 µm, representative image chosen from 2 independent experiments.

Several studies have shown that the phosphorylation state of AQP2 influences not only the targeting to the apical plasma membrane but also the stability and degradation of the AQP2^42^. We have recently shown that dbcAMP treatment prevents lysosomal degradation of AQP2^34^. Subsequently, we raised the question if the disappearance of AQP3 can be prevented by the activation of PKA signaling. To test this we performed the same setting as shown in Fig. 6 and treated the cells with dbcAMP during migration and checked if this had an influence on AQP3 localization. We observed, as shown in Fig. 8 that the treatment with dbcAMP prevented the disappearance of AQP3 in the leading cells with AQP3 still being localized at the plasma membrane even at the leading edge.

### dbcAMP increases migration in cells cultivated at 600 mosmol/kg

**Figure 9:**
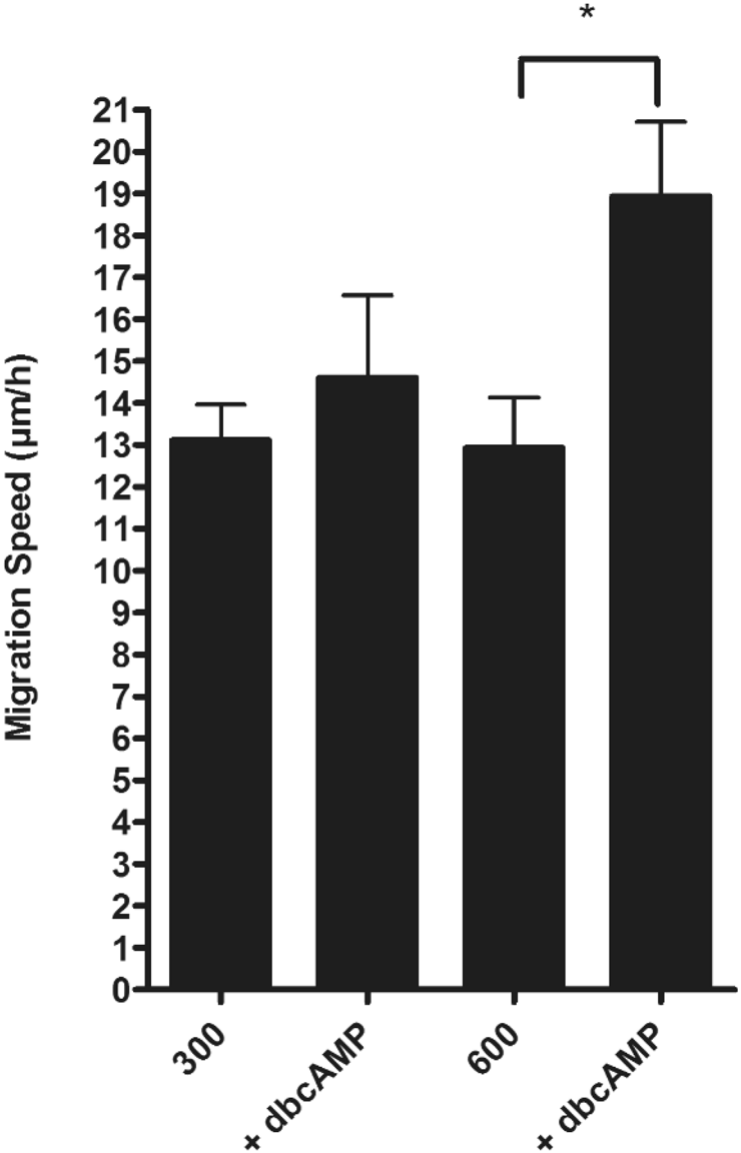
The treatment with dbcAMP induced the migration speed of the cells. Wound healing assays were performed with IMCD-cells cultivated at 300 or 600 mosmol/kg. A wound was induced migration of the cells were observed for 15 h. During this period of time one part of the cells were treated with dbcAMP (250 µM). The migration speed of the cells were calculated and significantly differences were calculated by ANOVA (p < 0.05, n ≤ 6).

Since dbcAMP forced the expression of AQP3 at the leading cells we tested if this has an influence on the migration speed of the cells. We repeated the wound healing assay and incubated the cells with dbcAMP during the migration process. The treatment with dbcAMP had no effect on cells which were cultivated at 300 mosmol/kg. In contrast, dbcAMP induced a significant increase of the migration speed in cells cultivated at 600 mosmol/kg (Fig. 9).

## Discussion

In our study we investigated the possible role of collecting duct aquaporins in cell migration using primary cultures of rat inner medullary CD. This is particularly interesting for its implication in the field of epithelial regeneration after kidney injury. The roles of aquaporins have been identified in several studies using different cell types^43^. At least 3 different AQPs are expressed in the principal cells of renal collecting duct. This unique expression pattern qualifies them as an ideal tool to analyze AQPs role on cell migration. So far a study using primary cultivated IMCD cells was missing. Therefore, this is the first study that used primary cultured IMCD cells to analyze the role of AQP on cell migration. Since the IMCD is faced with a unique osmotic environment, which is associated with extensive changes in gene expression^19^, we analyzed how hyper- and hypoosmolality affects cell migration. Although the used primary IMCD cell express endogenously AQP2–4, our experimental setting can only provide a simplified version of the situation in the collecting duct, where the cells are constantly challenged by osmotic differences on the apical and the basolateral membrane.

We found that the primary cultured IMCD cells are a suitable model due to the fact that they show a collective migration capability and due to the fact that they endogenously express AQP2–4. Moreover, the expression of AQP2–4 was induced under hyperosmotic conditions not only on the mRNA level but also on the protein level. Hyper osmolality induced massive changes in cell morphology including actin cytoskeleton rearrangement and beta catenin redistribution. Based on the postulated roles of AQPs in cell migration and the immense morphological changes, we expected differences in the migration behavior of cells cultivated at 300 vs. 600 mosmol/kg.

### Chronic hyper osmolality has no effect on migration speed

To our surprise, we did not observe significant differences in the migration speed between cells cultivated at 300 and 600 mosmol/kg. Hyper osmolality has many effects on cellular physiology. It can induce DNA damage, apoptosis, inhibit translation and transcription or induce oxidative stress^44^. It also induces changes in the organization of the actin cytoskeleton^44^. The type of reorganization depends on the analyzed cell type. All of the mentioned factors have a tremendous impact on the state of the cells. We also saw extensive differences on the cell morphology and the organization of the actin cytoskeleton. But all these changes had no effect on the migration capability of cells that were adapted to a given osmolality. On the other hand several factors that have been described to be associated with cell migration like AQP2–4 and NHE1 expression are induced by hyper osmolality^45,46^. We postulated that the pro-migratory factors (AQP2–4 or NHE1) and the anti-migratory factors (hyper osmolality induced stress) in adapted cells are in balance. A recent study showed that AQP2 modulates the activity of NHE1 and induces mechanical stability by inducing actin polymerization^47^. Since the expression of AQP2 is massively induced under hyperosmotic conditions this kind of interaction may be evident for cells cultivated at 600 mosmol/kg. We measured significant differences in migration speed when the cells were challenged by an acute change in osmolality. Consistent with previous observations, cell migration was impaired upon an acute increase by 300 mosmol/kg^48^. Probably in this case many of the mentioned processes were activated and the cells first had to get adapted to the hyper osmolality. Vice versa an acute drop of the osmolality by 300 mosmol/kg, which would be quite challenging for other cell types, was associated with a significant increase in the migration speed. In this case the pro-migratory factors mentioned above were still present but the anti-migratory factor hyperosmotic stress not, leading balance to the pro-migratory direction. We hypothesize that the hypoosmotic stimulus that promotes cell migration could be the low osmolality occurring during diuresis. It has been shown in the kidney that constant changes in cellular structure occur due to mechanical and chemical stimuli. When IMCD-cells were subjected to flow like it occurs during diuresis they showed altered actin cytoskeleton organization, which resulted in better viability^49^.

### Contribution of AQPs

It has been shown for AQP2–4 by many other studies that they can contribute to cell migration. The postulated role of AQPs in cell migration is that their expression at the leading edge facilitates the entry of water thereby pushing the lamellipodia forward^50^. For AQP2 it has been shown that it promotes cell migration by interacting with integrin β-1^9^. In primary cultured IMCD cells, as expected, AQP3 and AQP4 were expressed at the plasma membrane of the IMCD cells. The membrane expression of AQP2 was induced by dbcAMP as described by several other studies^41^. At the leading edge of migrating cells AQP3 expression was lost in the first 2–3 rows of cells and we did not observe any kind of accumulation in the lamellipodia of the cells. Several studies showed that AQP3 expression facilitates cell migration. The mentioned studies used either cells derived from AQP3 knockout mice, si- or shRNA mediated knockdown of AQP3 in or AQP3 overexpression cell lines. The localization of AQP3 was not studied in the majority of the studies. One study showed that in the MDA-MB-231 breast cancer cell line CXCL12 stimulation leads to an increased localization of AQP3 at the leading edge of the cell^51^. The loss of AQP3 expression within the first 2–3 rows of cells indicates that at least under physiological conditions AQP3 did not actively contribute to the migration. Intriguingly, a recent study reported that AQP3 expressing MDCK cells have a reduced collective migration capacity and that the contribution of AQP3 depends on the cell type^52^. AQP 3 is among the aquaglyceroporins that transport water and glycerol. Verkman et al. postulated that glycerol transport might be involved in ATP production mandatory for cell proliferation and wound healing^53^. We did not test the permeability of the IMCD-cells for glycerol and its role in cell migration. Since cell migration and cell proliferation are both very ATP-demanding processes in the cell, we cannot exclude that glycerol transport plays a role in the migration of IMCD-cells, especially because the enzyme glycerol kinase is strongly expressed in kidneys. Cell migration represents a wound healing process as a response to an injury. Hypoxic injury after ischemia–reperfusion in AQP3 deficient mice led to collecting duct abnormalities and significantly greater cell injury compared to AQP3 expressing mice^54^. This emphasizes the importance of AQP3 on recovery of the collecting duct after renal injury.

In contrast to AQP3, AQP4 was localized at the rear end of migrating cells. One potential explanation would be in concordance with the study by Stroka et al. that promoted the osmotic engine model^26^. According to their study water enters at the leading edge and exits the cell at the trailing edge. Thus, water could exit the cell at the trailing edge through AQP4. Although their study design included confined micro channels, it offers a possible explanation for the observed result. Other studies using kidney derived cells to analyze the contribution of AQP4 are missing. And even studies using endogenously AQP4 expressing cells are rare. The majority of the studies showed that AQP4 deficiency was associated with reduced migration capacity for example in astroglial cells or glioma cells and that high AQP4 expression was associated with tumor progression^55^. Also in glioma AQP4 was enriched in the plasma membrane^56^. At least seven isoforms of AQP4 have been identified so far^57^. The M1-AQP4, a long isoform with translation at first methionine and the M23-AQP4, a shorter isoform with translation methionine 23 are described as the two major isoforms^58,59^. The analyses of the M1 and M23 AQP4 isoform in astrocytes showed marked differences in the localization pattern; M1-AQP4 was enriched at the leading edge, while M23-AQP4 was localized at the rear end of the cells^60^.

### Effect of PKA signaling on cell migration

We observed that the stimulation of the protein kinase A (PKA) signaling accelerated the migration. But this was only the case when the cells were cultivated at 600 mosmol/kg. This indicates that the dbcAMP stimulation affects factors that are not present or active when cells were cultivated at 300 mosmol/kg. The trafficking of AQP2 to the apical plasma membrane is induced by the activation of PKA^33^. The expression of AQP2–4 was induced or elevated under hyperosmotic conditions^19^. We observed that dbcAMP led to accumulation of AQP2 at the leading edge of the cells. Moreover it prevented the degradation of AQP3 in the first rows of cells and promotes its localization at the leading edge. Therefore, the observed increase in migration upon dbcAMP might at least partially be promoted by AQP2 and AQP3. Arginine vasopressin (AVP) also increases the protein expression of AQP3^15^. As mentioned before AVP acts via the cAMP/PKA signaling pathway and its release from the posterior pituitary gland is stimulated by hyper osmolality, hypotension, and hypovolemia. The serum concentrations of AVP increase rapidly during acute hemorrhage, a situation during which the kidneys are prone to hypoxic cell injury^61^. Accordingly, our findings of increased cell migration upon dbcAMP stimulation offer a potential mechanism how wound healing in the collecting duct is augmented during acute shock states and that AQP 2 or AQP 3 could promote this process. Unfortunately, the primary cultured IMCD cells are not capable for genetic manipulation to induce a knockdown or knockout suing siRNA or CRISPR/Cas9 that would allow analyzing the contribution of AQP2–4 on cell migration. We are also fully aware that dbcAMP treatment influences other factors besides AQP2 and AQP4 such as the exchange protein directly activated by cAMP (EPAC) or the cytoskeleton just to mention two examples. While EPAC1 overexpression induced a reduction of bladder cancer cell migration^62^, the cAMP signaling induced migration in mouse embryonic stem cells^63^.

## Conclusions

Taken together, we showed that primary cultured IMCD cells display a collective cell migration behavior, which most likely takes place in response to kidney injury. Recent in vivo studies are consistent with our findings, showing that collective cell migration occurs after kidney epithelial injury^23^. Hyperosmotic stress had a major impact on cell morphology but not on migration speed. An acute change to a lower osmolality, which physiologically occurs in the kidney during diuresis, increased the cell migration speed markedly. Hyperosmolality played a role upon dbcAMP stimulation though, where it led to an increase in cell migration and restored AQP3 at the leading edge. AVP release, which acts via the cAMP/PKA pathway, is stimulated during hyperosmolality and acute hemorrhage. In these situations the principal cells are prone to possible injury. Thus, we hypothesize that AQP mediated cell migration in the CD occurs primarily under pathophysiological conditions. During an acute decrease of osmolality in diuresis the CD cells show an increased ability to adapt to possible cell injury by mechanism that are not primarily influenced by AQPs. Further studies, for example use of AQP2–4 deficient mice/cells, are needed to strengthen our findings of AQP contribution during migration in primary cultured IMCD cells.

## Supplementary Information

Supplementary Video 1.

Supplementary Video 2.

Supplementary Video 3.

Supplementary Video 4.

Supplementary Information.
