# Supplementary material for: Unexpected localization of AQP3 and AQP4 induced by migration of primary cultured IMCD cells
Source: Sci Rep. 2021 Jun 7;11:11930. doi: 10.1038/s41598-021-91369-y (PMC8185088; doi:10.1038/s41598-021-91369-y)
Supplement: Supplementary file 5 — Supplementary Information. [file 41598_2021_91369_MOESM5_ESM.docx]

Supplemental Figures

Unexpected localization of AQP3 and AQP4 induced by migration of primary cultured IMCD cells

Ralph Rose 1, Björn Kemper2, Albrecht Schwab 3, Eberhard Schlatter 1 and Bayram Edemir1,4,*

**S-Figure 1: Uncropped image of the AQP2-4 Western blot with GAPDH from figure 1**. The membranes were cut prior hybridization with the antibodies. The first antibody was stripped and the membrane reprobed with an anti GAPDH antibody. Full length membranes and raw images can be found as supplemental figure 12 (for AQP2 and GAPDH) and supplemental figure 13 (for AQP3, AQP4 and GAPDH).


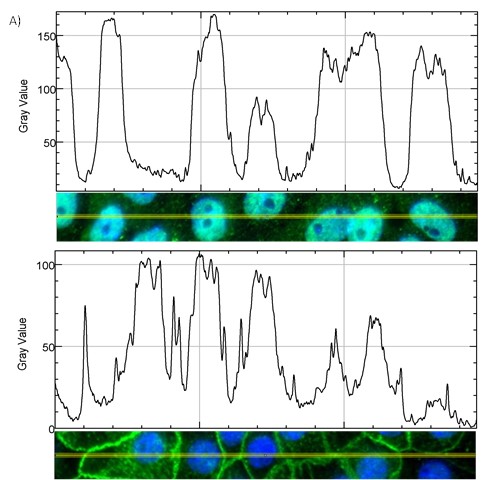

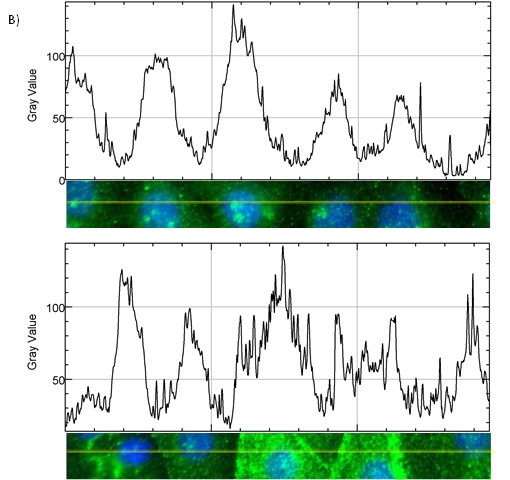


**S-Figure 2: Profile plots of signal intensities of AQP3 and AQP4 from figure 2.** Profile plots were generated using Fiji image analysis software to visualize signal distribution of AQP3 (A) and AQP4 (B) from figure 2. These results show enrichment of AQP3 and AQP4 expression at the cell-cell contacts.

**S-Figure 3: Hypertonicity induces morphological changes.** IMCD-cells were cultivated at 300 or 600 mosmol/kg. A scratch was induced and the cells were further cultivated for four hours. The cells were used to stain the actin cytoskeleton and beta tubulin. The cells were incubated with Alexa-488 labeled phalloidin (actin staining) or with a primary anti-tubulin antibody followed with Alexa-594 labeled second antibody. Different Z-sections are presented here. Images were taken from the leading edge of migrating cells Scale bar, 20 μm.


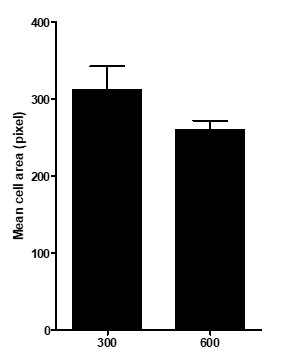


**S-Figure 4: Hypertonicity has no effect on area covered by a cell.** The covered area by cell was calculated using Fiji image analysis software. The Zo1 signals from figure 3 were used as an indirect factor for the covered area. Statistical analysis using unpaired t-test showed no statistically significant differences.


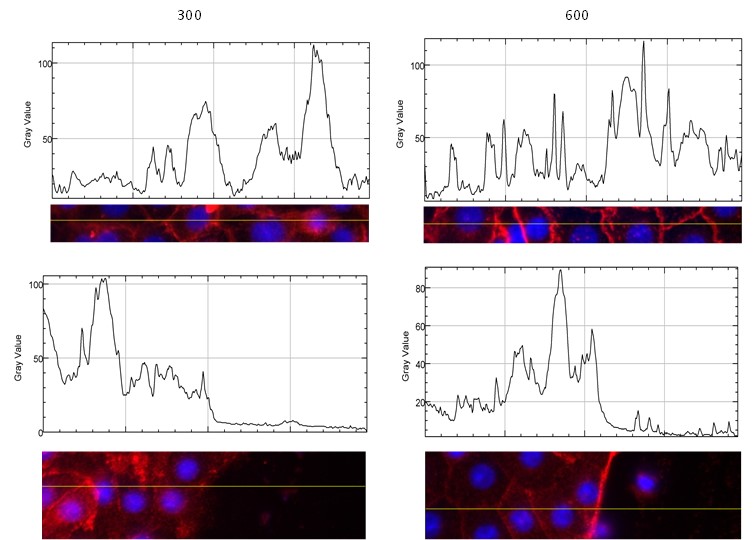


**S-Figure 5: Profile plots of signal intensities of Nhe1 from figure 5.** Profile plots were generated using Fiji image analysis software to visualize signal distribution of Nhe1 in cells cultivated at 300 or 600 mosmol/kg from figure 5.

A)

B)

**S-Figure 6: Hyperosmolality increases the protein expression of Nhe1 in IMCD-cells.** IMCD-cells have been cultivated at 300- and 600 mosmol/kg four six. Total protein was isolated and the NHE1 protein expression was analyzed using a specific antibody by Western blot. The expression of Gapdh served as loading control (A). The signals were densitometrical analyzed and the expression compared to 300 mosmol/kg was calculated. All values are means ± SEM (n=6). (B) Full length membrane. The membrane was cut before hybridization with the first antibody. After stripping the membrane was reprobed with an GAPDH antibody.


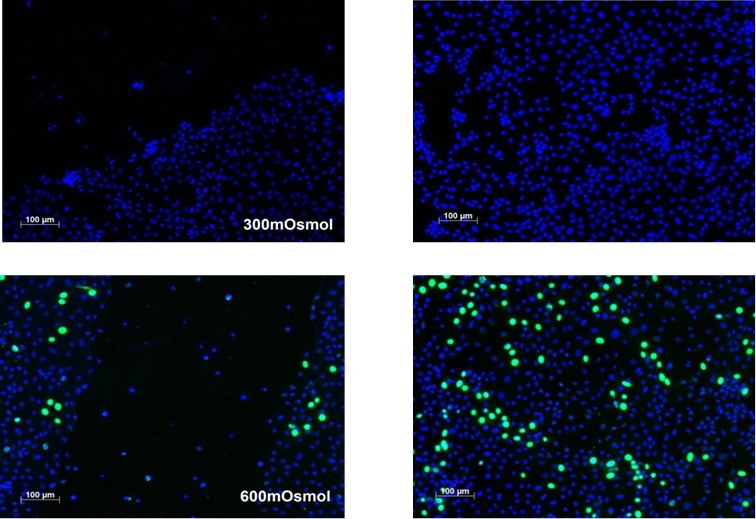


**S-Figure 7: BrdU-Assays of migrating IMCD-cells at different osmolality.** IMCD-cells were cultured at 300- and 600 mosmol/kg. After a scratch was made a BrdU-Assay was conducted using the BrdU-labeling kit by Roche®. The upper panel displays the cells at 300 mosmol/kg, the bottom one at 600 mosmol/kg. The cell nucleus is stained with DAPI (blue). The incorporated BrdU is indicated in green. Images were taken from the edge and the inside of the cell-monolayer.

**S-Figure 8: Calculation of covered cell area and cell volume in migrating cells using** **Quantitative phase imaging-based cell analysis with digital holographic microscopy.** IMCD-cells were cultured at 300- and 600 mosmol/kg. After a scratch was made digital holograms of the wound areas were recorded. The cell volume of the cells ate the front and the covered area over the time was calculated. Significant differences to time point 16 min (after initiation of scratch) are marked with *. n= 10-12 cells.


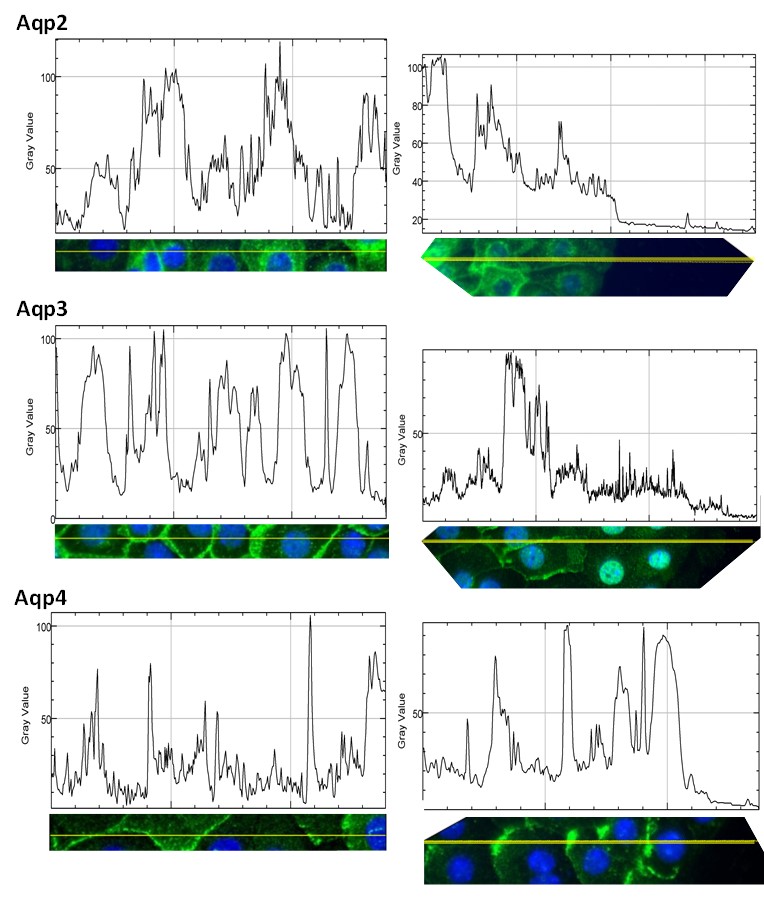


**S-Figure 9: Profile plots of signal intensities of AQP3 and AQP4 from figure 2.** Profile plots were generated using Fiji image analysis software to visualize signal distribution of AQP3 (A) and AQP4 (B) from figure 2. These analyses show no enrichment of either AQP3 or AQP4 at the leading edge of the migrating cells.


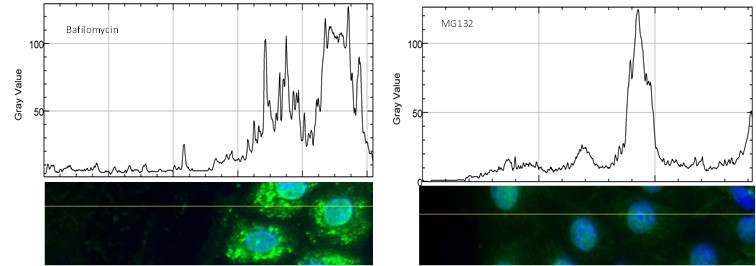


**S-Figure 10: Profile plots of signal intensities of AQP3 at leading edges of migrating cells.** Profile plots were generated using Fiji image analysis software to visualize signal distribution of AQP3 in cells treated either with bafilomycin or MG132 from figure 6.

**S-Figure 11: Bafilomycin treatment induces intracellular accumulation of AQP3.** IMCD cells were treated with bafilomycin (A) or MG132 (B) and localization of AQP3 was analyzed by immunofluorescence. LAMP1 antibody was used to stain lysosomal structures.

A)


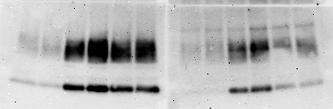


B)


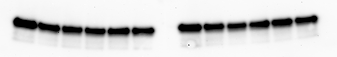


**S-Figure 12: Raw images showing complete membrane for figure 1.** A) The membranes were cut in pieces, and incubated with an anti-AQP2 antibody. B) After stripping of the AQP2 antibody the membranes were reprobed using an anti-GAPGH antibody.

**A)**


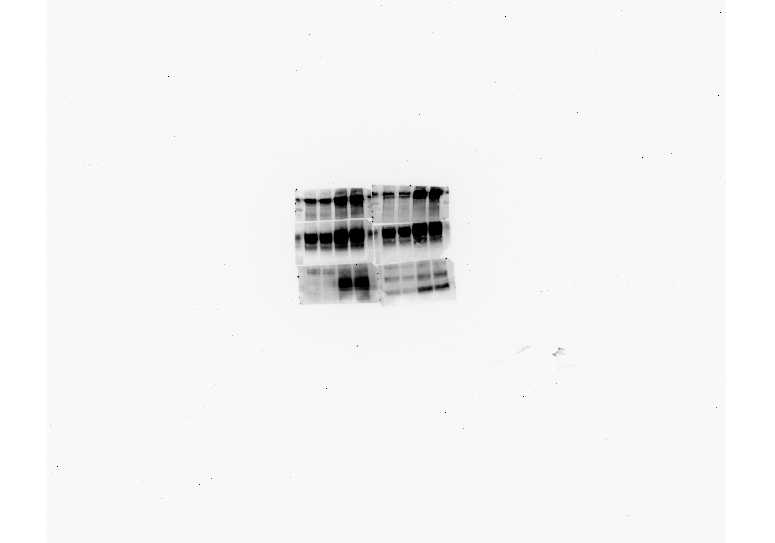


B)


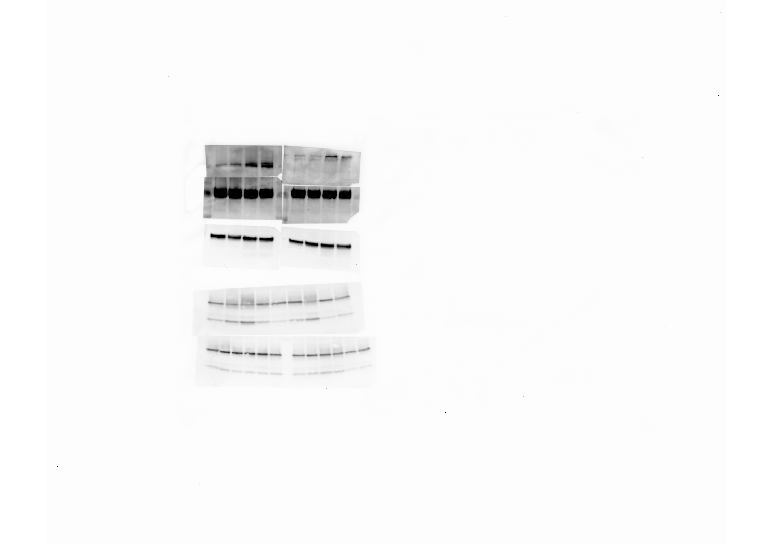


**S-Figure 13: Raw images showing complete membrane for figure 1 (AQP3 and 4) and supplemental figure 6 (NHE1) showing full membrane and GAPDH lower panel after stripping and reprobing).** A) The membranes were cut in pieces, and incubated with an anti-AQP3, AQP4 and NHE1 antibody. B) After stripping of the AQP2 antibody the membranes were reprobed using an anti-GAPGH antibody.
